# Early neonatal respiratory distress revealing meningitis caused by *Streptococcus pneumoniae* serotype 17F: a case report

**DOI:** 10.4314/ahs.v21i4.26

**Published:** 2021-12

**Authors:** Néhémie Nzoyikorera, Mouna Lehlimi, Idrissa Diawara, Khalid Zerouali, Raja Alami, Khalid Katfy, Fakhreddine Maaloum, Mounir Chemsia, Abderahim Habzi, Said Benomar, Naima Elmdaghri

**Affiliations:** 1 Faculty of Medicine and Pharmacy, Casablanca, Morocco, Hassan II University of Casablanca, Morocco; 2 Bacteriology-Virology and Hospital Hygiene Laboratory, Ibn Rochd University Hospital Centre, Casablanca, Morocco; 3 Service de néonatologie et de réanimation néonatale, hôpital d'enfants Abderrahim Harouchi, Centre Hospitalier Universitaire Ibn Rochd, Casablanca, Morocco; 4 Faculty of Sciences and Health Techniques, Mohammed VI University of Health Sciences (UM6SS) Casablanca, Morocco

**Keywords:** *Streptococcus pneumoniae*, neonatal meningitis, respiratory distress

## Abstract

**Background:**

*Streptococcus pneumoniae* (*S. pneumoniae*) is the first leading cause of invasive diseases such as meningitis, bacteremia and pneumoniae in children. In this case we report an early neonatal respiratory distress revealing meningitis caused by *S. pneumoniae* Serotype 17F through vertical transmission, in the newborn of 3 hours of live.

**Case description:**

A male late preterm newborn was born by vaginal delivery at a gestational age of 34 weeks. At 3 hours of life, he was admitted for early moderate neonatal respiratory distress in the Neonatal Medicine and Resuscitation Service. Cerebrospinal fluid culture yielded *S. pneumoniae* belonging to serotype 17F while the blood culture was negative. The same pneumococcal serotype was recovered from the high vaginal swab of the mother. Both isolates were found susceptible to all tested antibiotics except tetracycline and chloramphenicol to which the strain was resistant. Antibiotherapy management of the child included ceftriaxone at 150mg/kg/day for 21 days, in combination with gentamycin at 5 mg/kg/day for 5 days. ciprofloxacin was added at 40mg/kg/day in two doses for a period of three weeks as the baby presented a hydrocephalus.

**Conclusion:**

This finding shows that clinical manifestations of neonatal pneumococcal meningitis may be atypical and/or misleading.

## Background

*Streptococcus pneumoniae* (*S. pneumoniae*) is the first leading cause of invasive diseases such as meningitis, bacteremia and pneumonia in children^1^. However, this microorganism is still a rare cause of meningitis in newborns but it is related with high morbidity and mortality^2^. We present a case of early neonatal respiratory distress revealing meningitis caused by >*S. pneumoniae* through vertical transmission.

## Case description

A male late preterm newborn from an incompletely monitored pregnancy, was born by vaginal delivery at a gestational age of 34 weeks. Adaptation to extrauterine life was mild with an Apgar score at birth of 5/10 which required aspiration and oxygen therapy. Birth weight was 1900g and cranial perimeter was 31 cm. At 3 hours of life, he was admitted for early moderate neonatal respiratory distress at the Neonatal Medicine and Resuscitation Service of Abderrahim Harouchi Children Hospital in Casablanca, Morocco. The anamnesis was positive for maternal chorioamnionitis and spontaneous prematurity under 35 weeks. At initial physical examination, the newborn was hemodinamically stable, afebrile at 36.8°C, tachypneic at 72 cycles/minute, with room air oxygen saturation of 92% and a Silverman score of 5/10. Primitive reflexes were weak and the anterior fontanelle was normotensive. No malformations were found during this examination.

The initial chest radiography was normal. Cranial ultrasound showed a grade 4 intraventricular hemorrhage while the cerebral computed tomography revealed diffuse meningeal enhancement and minimal ventricular dilatation.

The lumbar puncture was done and the Cerebrospinal fluid (CSF) was sent to the microbiology laboratory of Ibn Rochd University Hospital Centre for analysis. Simultaneously, the blood culture was performed. CSF was hematic with uncountable white cells, 5.14g/L of protein, 0.19g/l of glucose and 122mEq/l of chlorure but the C-reactive protein (CRP) was negative at 2.2 mg/l. After 24 hours of incubation under 5% CO^2^, the bacteria grew on Chocolate agar. The culture on Columbia Nalidixic Acid agar (CNA) under 5% CO^2^ showed after 24h of incubation a pure culture of Gram-positive, lanceolate and encapsulated diplococci with negative catalase test. The isolate was soluble on 10% sodium desoxycholate; thus, it was confirmed as *S. pneumoniae*. The blood culture showed no growth of any bacteria.

Antibiotic susceptibility tests were performed on Mueller-Hinton agar additioned with 5% of sheep blood (BioMérieux, Marcy-l'Etoile, France) and interpreted according to the European Committee on Antimicrobial Susceptibility Testing (EUCAST 2018) recommendations^3^. The minimum inhibitory concentration (MIC) of penicillin G, ampicillin and ceftriaxone were determined by E-test method. Oxacillin, erythromycin, chloramphenicol, clindamycin, vancomycin, co-trimoxazole and levofloxacin were tested by disc diffusion. The strain was susceptible to penicillin G (CMI= 0.015mg/L), ampicillin (CMI= 0.016mg/L), ceftriaxone (CMI= 0.004mg/L) and all other antibiotics except tetracycline and chloramphenicol to which the strain was resistant.

As soon as the pneumococcus was identified, the vaginal swab from the mother was done and sent to the microbiology laboratory for microbial investigation. The pneumococcus grew after 24h under 5% CO2 on CNA agar with the same susceptibility pattern to different antibiotics tested on the strain isolated from the newborn. Serogrouping of the two strains was done by the checkerboard method with Pneumotest-latex (Statens Serum Institute antisera, Copenhague, Denmark) and serotyping was performed by multiplex PCR according to the procedures previously described by CDC^4^. These methods of serogrouping/serotyping classified the serotype of the 2 strains as 17F.

Antibiotherapy management of the neonatal child included a third-generation cephalosporin, ceftriaxone at 150mg/kg/day for 21 days, in combination with gentamycin at 5 mg/kg/day for 5 days. In the evolution, the newborn presented a hydrocephalus and ciprofloxacin was added at 40mg/kg/day in two doses for a period of three weeks. Clinical and biological evolution were favorable under treatment. The lumbar puncture done 8 days after admission was sterile. A control cerebral computed tomography revealed the presence of periventricular leukomalacia. A sleep electroencephalogram (EEG) showed diffuse waveforms associated with poorly organised sleep.

## Discussion

Neonatal meningitis is a devastating invasive disease associated with high mortality and morbidity with the overall mortality rate of neonatal meningitis ranging between 10% and 15%^2^. Group B Streptococcus agalactiae (GBS) is the well-known leading cause of neonatal meningitis followed by Escherichia coli K1 and Listeria monocytogenes^5^. *S. pneumoniae* is a rare but fatal agent of meningitis in the neonatal period. The isolation of *S. pneumoniae* as well as data on incidence of nasopharyngeal carriage in the neonatal period are rare. The bacteria were recovered from CSF and absent in the blood. Discordances between blood and CSF culture results have been reported. In up to a third of infants with bacterial meningitis, blood culture are negative, and this highlights the importance of CSF culture^6^.

In many cases, the source of pneumococcus in neonatal meningitis remains unknown. Two possible forms of transmission of this pathogen have been reported; horizontal due to local infections or colonization and vertical by vaginal colonization of the pneumococcus^7^. In our case, the same microorganism was isolated from the maternal high vaginal tract. In other hands, the strain isolated from the children and her mother belonged to the same serotype (17F) and showed the same antibiogram pattern. Thus, the vertical transmission was the most probable mode of transmission.

The recovery of *S. pneumoniae* from high vaginal swab was surprising as its ecological niche and reservoir is the human nasopharynx^8^. Incidence of vaginal colonization, often asymptomatic, is very low, varying between 0.03%–0.75% of cases^9^. Nevertheless, female genital tract infection may occur through four different possible mechanisms including infection via the vaginal primary resident flora from intra uterine device, the use of tampons, during orogenital sexual practices; via gastrointestinal tract; via lymphatics and via bloodstream^10^. In our case, the newborn mother presented only premature rupture of membranes at 34 weeks of gestational age. Some maternal predisposing risk factors such as early membranes rupture, endometritis and foul-smelling vaginal discharge have been reported for genital infections^11^.

In our case, the newborn presented pneumococcal meningitis. The clinical presentation of neonatal meningitis is similar to those of neonatal sepsis without meningitis^12^. In the large study on neonatal pneumococcal meningitis conducted by et Arfi et al. (2017), the authors found no clinical or biological characteristic that distinguished *S. pneumoniae* and GBS meningitis^13^. Indeed, our patient was hospitalised for respiratory distress. As reported elsewhere, the causes of respiratory distress in a newborn are diverse and multisystemic. The underlying cause varies and does not always lie within the lungs. The differential diagnosis of respiratory distress in the newborn include airway, pulmonary, thoracic, neuromuscular (eg meningitis) and others^14^. Based on these statement, clinical manifestations of neonatal pneumococcal meningitis may be atypical and/or misleading. Some pneumococcal serotypes such as serotype 1 and 5 are associated with invasive diseases in children, and are usually found from newborn invasive pneumococcal diseases^12^. In the study of Gardien et al. (2001) on the epidemiology of pneumococcal infections of the genital tract of women, one-third of the strains belonged to serotype 3; followed by serogroup/serotype 19,1,9 and 1113. In our case, the strain isolated from the newborn and her mother belonged to the serotype 17F. To our knowledge, this case is the first well documented case of neonatal meningitis caused by *S. pneumoniae* serotype 17F.

In every neonatal early-onset meningitis suspicion case, rapid empiric and aggressive antibiotic treatment is initiated especially with large broad-spectrum molecules targeting all suspected and possible meningitis bacterial agents^11^. Therapeutic management by using the combination of amoxicillin with a third-generation cephalosporin and an aminoglycoside is generally recommended in early-onset neonatal meningitis^14^.

This treatment could be modified according to the germ isolated and its antimicrobial susceptibility pattern. In our case, the antibiotherapy included a combination of a third-generation cephalosporin (ceftriaxone) for 21 days combined with gentamycin for 5 days. The therapeutic efficacy is judged based on the clinical evolution but especially on the sterility of the control CSF recommended 48 hours after the beginning of the treatment^15^. The systematic association with a fluoroquinolone (ciprofloxacin in particular) on the initial antibiotherapy during the first 4 to 5 days of treatment allows the reduction of the frequency of immediate complications^15^; thus, in our case ciprofloxacin was used for 21 days as there was hydrocephalus.

In meningitis cases, long-term complications are observed among 20–50% of survivors, depending on time of diagnosis and therapy and virulence of the infecting pathogen^12^. In the study conducted by Bekiesińska-Figatowska et al.^19^, they have the follow-up data only in 7 out of 10 patients (3 lost from follow-up). Their results in terms of lifelong sequelae were dramatic and showed 1 death, various degree of paresis in 6 patients, numerous neurosurgical procedures in 3, epilepsy in 2, severe intellectual disability in 2 and delayed psychomotor development in 1. Those results highlight the need of long-term follow-up of meningitis cases.

## Conclusion

Clinical manifestations of neonatal pneumococcal meningitis may be atypical and/or misleading. Screening for carriage or infection of *S. pneumoniae* in pregnant women in the female genital tract should be initiated for appropriate management of the mother and the newborn. A lumbar puncture for microbiological analysis should be done systematically for any newborn with toxic appearance. Finally, we insist on the need to follow these children for several years to accurately assess the neurosensory sequelae.

## References

[1] Henriques-Normark B, Tuomanen EI (2013). The Pneumococcus: Epidemiology, Microbiology, and Pathogenesis. Cold Spring Harbor Perspectives in Medicine.

[2] Gaschignard J, Levy C, Romain O, Cohen R, Bingen E, Aujard Y (2011). Neonatal Bacterial Meningitis: 444 Cases in 7 Years. Pediatr Infect Dis J.

[3] CASFM / EUCAST (2018). Streptococcus pneumoniae. Société Française de Microbiologie.

[4] Meningitis | Lab Manual | Characterization - Molecular Typing | CDC.

[5] Arfi A, Cohen R, Varon E, Béchet S, Bonacorsi S, Levy C (2017). Case-control study shows that neonatal pneumococcal meningitis cannot be distinguished from group B Streptococcus cases. Acta Paediatrica.

[6] Smith P, Garges H, Cotton C, Walsh T, Clark R, Benjamin D (2008). Meningitis in Preterm Neonates: Importance of Cerebrospinal Fluid Parameters. American Journal of Perinatology.

[7] Barichello T, Fagundes GD, Generoso JS, Elias SG, Simoes LR, Teixeira AL (2013). Pathophysiology of neonatal acute bacterial meningitis. Journal of Medical Microbiology.

[8] Simell B, Auranen K, Käyhty H, Goldblatt D, Dagan R, O'Brien KL (2012). The fundamental link between pneumococcal carriage and disease. Expert Review of Vaccines.

[9] Hermoso Torregrosa C, Castillo MCZ y MT (2012). Streptococcus pneumoniae: An Unusual Pathogen in Neonatal Sepsis of Vertical Transmission. Archivos de Bronconeumología (English Edition).

[10] E DrM, Kuruvilla DTS, Chandrakar DS (2015). Streptococcus pneumoniae: An Unusual Pathogen Seen In High Vaginal Swab. International Journal of Biomedical Research.

[11] Gordon SM, Srinivasan L, Harris MC (2017). Neonatal Meningitis: Overcoming Challenges in Diagnosis, Prognosis, and Treatment with Omics. Front Pediatr.

[12] Şah İpek M (2019). Neonatal Bacterial Meningitis.

[13] Arfi A, Cohen R, Varon E, Béchet S, Bonacorsi S, Levy C (2017). Case-control study shows that neonatal pneumococcal meningitis cannot be distinguished from group B Streptococcus cases. Acta Paediatr.

[14] Reuter S, Moser C, Baack M (2014). Respiratory distress in the newborn. Pediatr Rev.

[15] Assandri E, Amorín B, Gesuele JP, Algorta G, Pírez MC (2015). Enfermedad neumoccócica invasora en recién nacidos, antes y después de la vacunación universal con vacuna conjugada 7 y 13 valente en Uruguay. Rev chil infectol.

[16] Gardien E, Schlegel L, Grégory A, Tognelli S, Frémaux A, Geslin P (2001). À propos d'un cas de salpingite à Streptococcus pneumoniae, épidemiologie des infections génitales de la femme à pneumocoque. Pathologie Biologie.

[17] Gaschignard J, Levy C, Romain O, Cohen R, Bingen E, Aujard Y (2011). Neonatal Bacterial Meningitis: 444 Cases in 7 Years. The Pediatric Infectious Disease Journal.

[18] Berardi A, Lugli L, Rossi C, China M, Vellani G, Contiero R (2010). Neonatal bacterial meningitis. Minerva Pediatrica.

[19] Bekiesińska-Figatowska M, Duczkowska A, Duczkowski M, Brągoszewska H, Mądzik J, Iwanowska B (2020). Pneumococcal Meningitis and Its Sequelae - A Devastating CNS Disease. J Mother Child.

